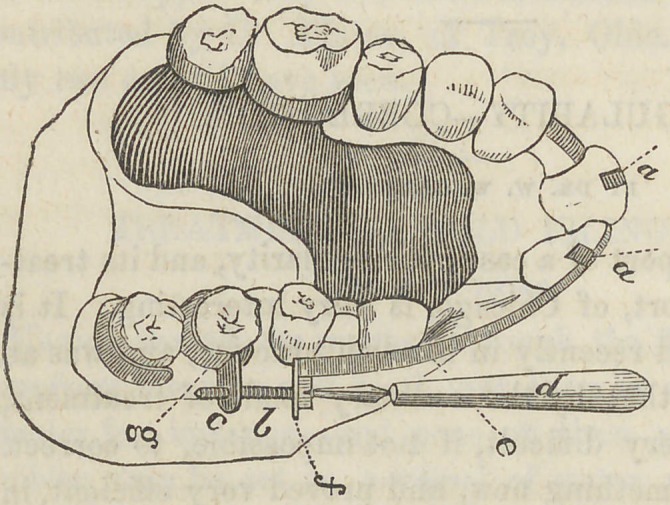# Irregularity—Correction

**Published:** 1857-09

**Authors:** W. W. Allport


### IRREGULARITY—CORRECTION.

BY DR. W. W. ALLPORT.

The following report of a case of irregularity, and its treatment, by Dr. Allport, of Chicago, is very interesting. It is a case that occurred recently in his own practice, and was an extreme case, one that by the ordinary mode of treatment, would have been very difficult, if not impossible, to correct. The appliance is something new, and proved very efficient, in a comparatively short time. The teeth now occupy a good position. The operation produced an entire change upon the face of the patient. The following description of Dr. A.'s is very clear and concise, and by the aid of the cuts, will be at once comprehended.—ED.

Fig. 1 represents the teeth as in their original position.

Fig. 2 represents the teeth at the expiration of five weeks after the apparatus was applied.

Fig. 3 represents the plate, clasped to two molars, on each side, with the band extending around the outside, and to which are soldered two hooks (*a a*) passing over the ends of the incisors, to prevent the band slipping too high upon the teeth.

To each end of the band is attached a screw (*b*) passing through a nut (*c*) soldered to the clasp, and by means of which the band was tightened every other day, on each side of the mouth, with a screw-driver (*d*).

This process was continued for five weeks; the whole was then allowed to remain without further tightening for two weeks longer, when the apparatus was removed.

To retain the teeth in this position, a plate was again attached to two molars, with narrow strips of gold soldered to it, and passing over the inside, and hooked to the ends of the incisor teeth.

During the whole process, the patient made but very little complaint of soreness, or of inconvenience from the apparatus. The patient was a lady twenty-two years of age.

## Figures and Tables

**Fig. 1 f1:**
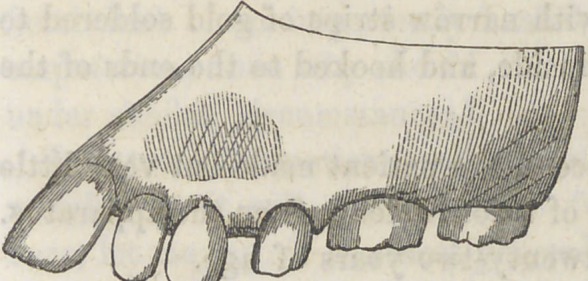


**Fig. 2 f2:**
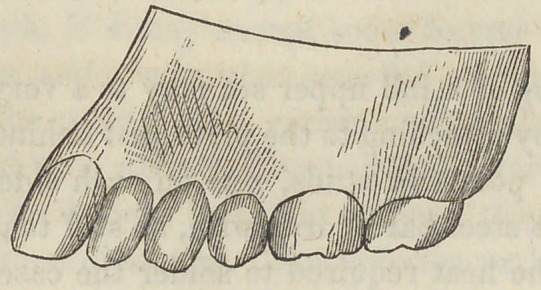


**Fig. 3 f3:**